# Eruptive Granuloma Annulare in an Elderly Man With Diabetes

**DOI:** 10.7759/cureus.21242

**Published:** 2022-01-14

**Authors:** Mohammed A Al Ameer, Sahar H Al-Natour, Hassan Ahmed A Alsahaf, Ghada Omar Alakloby, Jamal M Alqahtani

**Affiliations:** 1 Department of Dermatology, King Fahad Hospital Hofuf, Al-Ahsa, SAU; 2 Department of Dermatology, College of Medicine, Imam Abdulrahman Bin Faisal University, Dammam, SAU; 3 Internal Medicine, Johns Hopkins Aramco Healthcare (JHAH), Dhahran, SAU

**Keywords:** necrobiosis, palisading, pruritus, diabetes, granuloma annulare

## Abstract

Although the etiology of granuloma annulare (GA) remains unknown, it has been associated with many reported triggers, including chronic medical illnesses and malignancy. Herein, we present a case of an elderly man with diabetes who has a generalized variant of granuloma annulare. The patient had a six-month history of multiple skin-colored, scattered, itchy papules disseminated over the trunk. Clinical features with histological findings are described. Therefore, this case report aimed to present a rare type of diffuse, eruptive granuloma annulare.

## Introduction

Granuloma annulare (GA) is characterized by cutaneous granulomatous inflammatory dermatosis with a self-limited benign course. However, its etiology remains undetermined, and the pathogenesis is poorly understood with variable clinical duration. Most of the reported cases of GA are sporadic. Several morphological forms and clinical subtypes have been described in the literature, including generalized, localized, subcutaneous, and perforating GA. It can affect any part of the skin, usually presents as a group of papules in a symmetrical pattern with an annular configuration, and is histologically characterized by necrobiosis surrounded by lymphohistiocytic infiltrates in the dermis or subcutaneous tissue layer of the skin [1].

The generalized form of GA comprises 8-15% of cases. As most of the patients are in the adult age group, it can also affect children and younger age groups. In contrast to localized disorders, the trunk, as well as the neck and extremities, is usually implicated. The scalp, face, hands, and soles are also all affected. Widespread papules, consolidated to manifest as small annular plaques or larger discolored patches with elevated arcuate and serpiginous borders, indicate generalized GA [2].

## Case presentation

**Figure 1:**
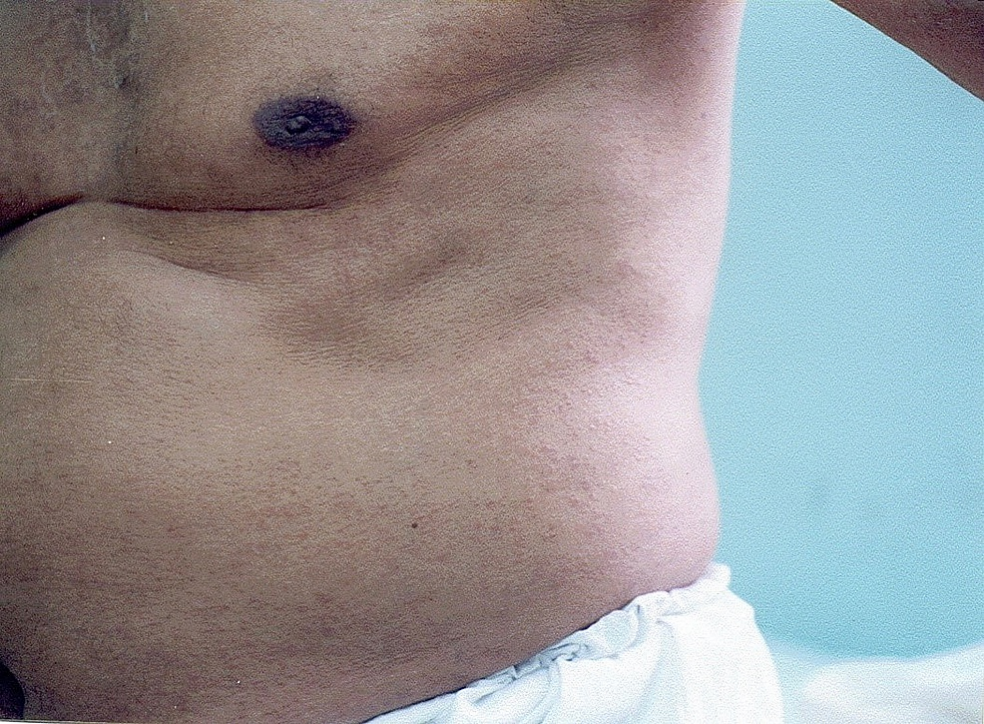
Multiple skin-colored to faint erythematous papules on the trunk.

A 70-year-old man visited our institution with a six-month history of multiple diffused itchy papules of skin color on his trunk. He had a history of longstanding uncontrolled type 2 diabetes mellitus (DM) actively managed with insulin therapy and hypoglycemic agents. On examination, he had multiple scattered dispersed skin-colored to faint erythematous papules, 0.5-1 cm in size, mainly on the upper part of the chest, abdomen, proximal aspects of arms, and thighs (Figures 1).

**Figure 2:**
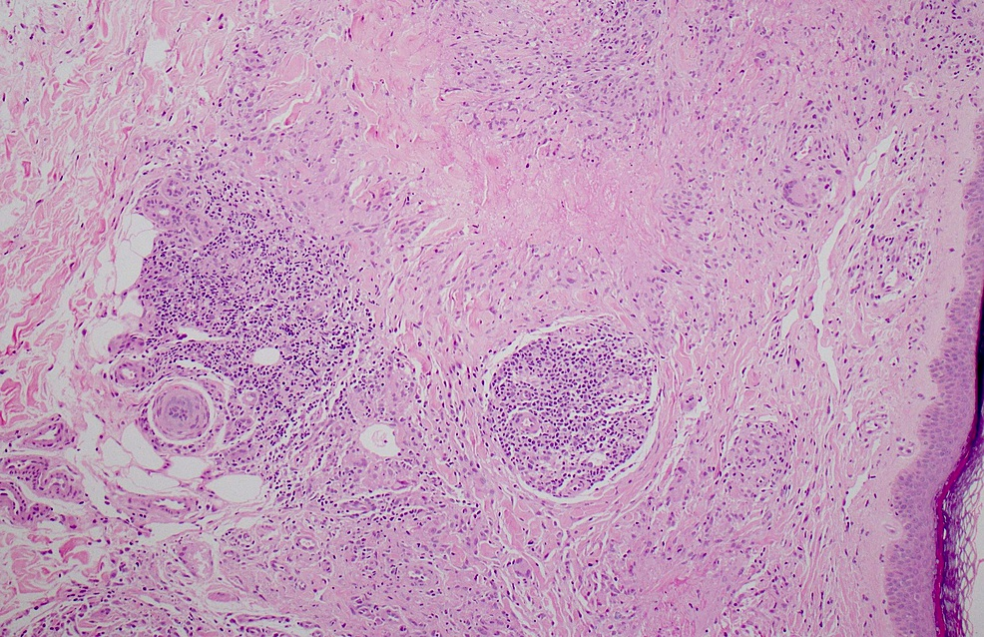
Low-power photomicrograph showing palisading granulomatous inflammation with central necrobiosis in mid-reticular dermis, including rare multinucleated giant cells.

**Figure 3:**
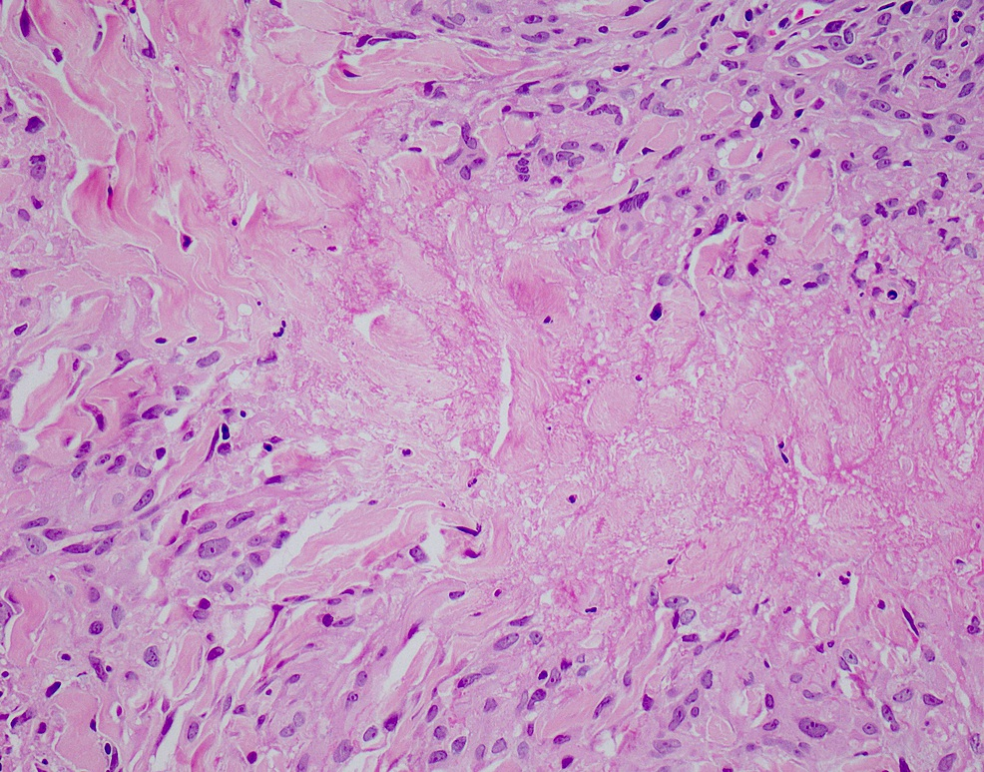
High-power photomicrograph showing palisading lymphohistiocytic infiltrate, surrounding central necrobiosis.

The oral mucosa was not involved. Laboratory investigations showed objective evidence of microcytic hypochromic anemia with a hemoglobin of 8.2 g/dl (average: 11-16 g/dl). The blood glucose level obtained during the fasting state was 182 mg/dl, and the HbA1c was 7.9%. The renal and liver panels, fasting lipid profile, thyroid function test, antinuclear antibodies, urinalysis, and chest radiograph were unremarkable. On histopathological examination under the microscope, a palisading pattern with granuloma located in the mid-reticular dermis surrounding necrobiotic collagen with prominent mucin deposition was revealed (Figure 2). A semicircular palisade of lymphohistiocytic cells with giant cells formed the granuloma (Figure 3). The patient was diagnosed with generalized variant GA based on the correlation of clinical evaluation with histopathological findings. The patient has been managed with narrowband ultraviolet B (UVB) of 180 millijoules/cm^2^ twice weekly and oral antihistamine once daily (cetirizine, 10 mg HS); most lesions have regressed and improved after three months.

## Discussion

The underlying cause of GA remains undetermined. Its triggering factors include insect stings, human immunodeficiency virus, Herpes zoster, and Epstein-Barr virus, which are all viral infections associated with GA. Allopurinol and gold, along with other drugs, have been effective for their treatment. Although its association with DM is debatable, some studies have proposed that it might be associated with DM, as described in our case [3]. An underlying paraneoplastic process should be considered as an underlying etiology behind GA, especially in lymphomas [4].

The main histopathological finding of the GA is a granuloma with lymphohistiocytic infiltrate and altered collagen tissue with mucus deposition. The inflammatory infiltrates might be palisaded and organized in an interstitial pattern or a mixture of both. The classical appearance of GA is represented either in a single or several foci of inflammation, with a central core of disorganized collagen (necrobiosis) bordered by a wall of palisaded histiocytes. The necrobiotic centers are usually round, nucleolus-free, and slightly basophilic, with a loss of collagen bundle definition and few or no elastic tissue fibers [1].

Various treatment modalities have been attempted. However, GA has the likelihood of resolving on its own, making the treatment efficacy assessment difficult and biased [5]. Many practicing dermatologists utilize potent topical steroids with or without occlusion; however, they are usually ineffective. Moreover, potassium iodide has been shown to have a positive effect in some studies that treated generalized variants of GA [6]. Photochemotherapy also appears to be an effective modality in the case of generalized GA, and good results have been reported using both oral and topical psoralens [7]. Furthermore, a few reports have used cyclosporin to treat generalized GA with good results. Narrowband UVB has also been found to be an effective treatment for generalized GA, as in our patient [8]. Rapid resolution of recalcitrant disseminated lesions has been reported with the use of infliximab [9].

## Conclusions

Generalized GA is the least prevalent type of GA case. Although most patients are middle-aged, it can also occur in younger age groups and in children. In contrast to localized GA, the trunk, neck, and extremities are commonly implicated. Because GA has the propensity to resolve spontaneously, determining the effectiveness of any treatment is complicated. Therefore, a more significant number of properly designed multiple randomized control trials would be recommended.
